# Efficacy of 3D screens for sustainable mosquito control: a semi-field experimental hut evaluation in northeastern Tanzania

**DOI:** 10.1186/s13071-023-06032-4

**Published:** 2023-11-15

**Authors:** Subam Kathet, Wema Sudi, Victor Mwingira, Patrick Tungu, Mikko Aalto, Tomi Hakala, Markku Honkala, Robert Malima, William Kisinza, Seppo Meri, Ayman Khattab

**Affiliations:** 1 Department of Bacteriology and Immunology, Haartman Institute, and Translational Immunology Research Program, University of Helsinki, 00014 Helsinki, Finland; 2 Amani Medical Research Centre, National Institute for Medical Research, Muheza, Tanzania; 3 East Africa University, Bosaso, Somalia; 4 Department of Materials Science, Tampere University of Technology, P.O. Box 589, 33101 Tampere, Finland; 5 HUSLAB Diagnostic Center, Helsinki University Central Hospital, N00029 Helsinki, Finland; 6 Department of Nucleic Acid Research, Genetic Engineering and Biotechnology Research Institute, City of Scientific Research and Technological Applications, New Borg El-Arab City, 21934 Alexandria Egypt

**Keywords:** Malaria, *Anopheles*, Mosquito control, Window double screens, 3D-Screens, 3D-WDST, Experimental huts, Muheza, Northeastern Tanzania

## Abstract

**Background:**

A three-dimensional window screen (3D-Screen) has been developed to create a window double-screen trap (3D-WDST), effectively capturing and preventing the escape of mosquitoes. A 2015 laboratory study demonstrated the 3D-Screen's efficacy, capturing 92% of mosquitoes in a double-screen setup during wind tunnel assays. To further evaluate its effectiveness, phase II experimental hut trials were conducted in Muheza, Tanzania.

**Methods:**

Three experimental hut trials were carried out between 2016 and 2017. Trial I tested two versions of the 3D-WDST in huts with open or closed eaves, with one version using a single 3D-Screen and the other using two 3D-Screens. Trial II examined the 3D-WDST with two 3D-Screens in huts with or without baffles, while Trial III compared handmade and machine-made 3D structures. Mosquito capturing efficacy of the 3D-WDST was measured by comparing the number of mosquitoes collected in the test hut to a control hut with standard exit traps.

**Results:**

Trial I showed that the 3D-WDST with two 3D-Screens used in huts with open eaves achieved the highest mosquito-capturing efficacy. This treatment captured 33.11% (CI 7.40–58.81) of female anophelines relative to the total collected in this hut (3D-WDST and room collections) and 27.27% (CI 4.23–50.31) of female anophelines relative to the total collected in the control hut (exit traps, room, and verandahs collections). In Trial II, the two 3D-Screens version of the 3D-WDST captured 70.32% (CI 56.87–83.77) and 51.07% (CI 21.72–80.41) of female anophelines in huts with and without baffles, respectively. Compared to the control hut, the capturing efficacy for female anophelines was 138.6% (37.23–239.9) and 42.41% (14.77–70.05) for huts with and without baffles, respectively. Trial III demonstrated similar performance between hand- and machine-made 3D structures.

**Conclusions:**

The 3D-WDST proved effective in capturing malaria vectors under semi-field experimental hut conditions. Using 3D-Screens on both sides of the window openings was more effective than using a single-sided 3D-Screen. Additionally, both hand- and machine-made 3D structures exhibited equally effective performance, supporting the production of durable cones on an industrial scale for future large-scale studies evaluating the 3D-WDST at the community level.

**Graphical Abstract:**

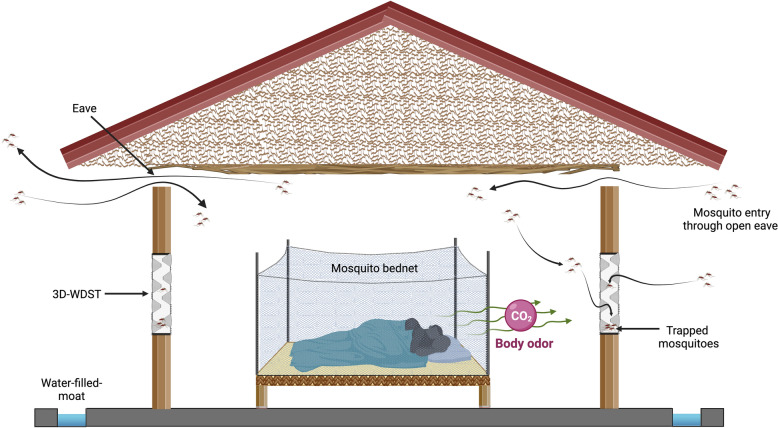

## Background

Malaria is a vector-borne infection caused by *Plasmodium* parasites and transmitted to humans through the bite of female *Anopheles* mosquito [1]. It is endemic in several tropical and sub-tropical countries, and it is one of the major causes of mortality and morbidity especially among children under 5 [1, 2]. The rate of malaria transmission from vector to host largely depends on mosquito host-seeking behavior, its feeding preferences, and resting behavior. While major African vectors such as *Anopheles gambiae* s.s. (sensu stricto), *An. funestus* s.s. [3, 4], and Asian vector *An. stephensi* are endophagic (feeds indoor) in nature, vectors like *An. arabensis* from the same species complex (*An. gambiae* sensu lato) as *An. gambiae* s.s. exhibit both endophagic and exophagic (feeds outdoor) behavior [5, 6]. Similarly, *An. gambiae* s.s. and *An. funestus* s.s. mostly exhibit endophilic behavior where they spend a considerable amount of time indoors post blood meal; however, some studies have reported exophilic behavior as well [4, 7]. *Anopheles arabensis*, on the other hand, exhibits exophilly [8, 9] with some studies reporting both endophilic and exophilic (ambivalent resting behavior) nature [10, 11]. Exophagic and exophilic mosquitoes are best controlled through the elimination of breeding sites while endophilic mosquitoes can be controlled by indoor residual insecticide spraying (IRS). Malaria control methods that minimize human-vector contact such insecticide-treated bed nets (ITNs) and house-proofing are best suited for endophagic mosquitoes.

ITNs and IRS are major vector control approaches currently under the WHO recommended guidelines and are included in most large-scale malaria vector control campaigns. Although the use of long-lasting ITNs was a great achievement in minimizing human-mosquito contact whereby regular mosquito nets are subjected to insecticide treatment and the emission takes place slowly during the lifespan of a net, the extensive use of malaria control measures, based on insecticides, has resulted in the emergence of insecticide-resistant mosquitoes [12, 13], which now poses a significant threat to the global malaria control campaign. In this challenging scenario, an efficient, environment friendly and more sustainable approach to reduce malaria vectors which is non-reliant on insecticides is highly sought. To address this gap, we developed a novel type of mosquito screen, the 3D-Screen, which can allow mosquitoes to penetrate only one side of the screen, i.e. the permissive side [14]. The 3D-Screen can be used parallel to a traditional screen or a second 3D-Screen to create a window double-screen trap setup. This double-screen setup creates an effective window trap that can use host-seeking activity to capture both endophagic and endophilic mosquitoes.

In a laboratory phase study [14], we developed several 3D-Screen prototypes and tested them for their efficacy to capture mosquitoes during their host-seeking activity. The cone-shaped prototype stood out to fulfill the desired target product profile, i.e. efficacy, durability, ease of production, and low cost. In wind tunnel experiments, the cone-based 3D-Screen captured 92% of the mosquitoes released in the tunnel in a double-screen setup. No mosquitoes could escape the double 3D-Screen trap once they penetrated the permissive side of the 3D-Screen. Thus, the cone-based 3D-Screen effectively acted as a unidirectional mosquito screen. In this experimental hut study, we explored the potential of the cone-based 3D-Screen installed on windows to form a 3D Window Double Screen Trap (3D-WDST) under different test conditions to capture the malaria mosquitoes.

## Methods

### The 3D window double-screen design

**Fig. 1:**
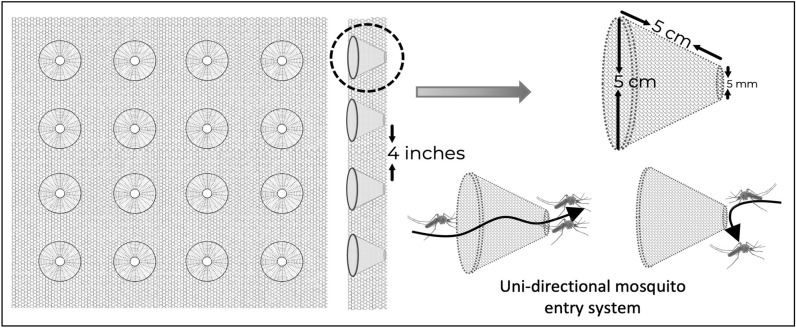
Three-dimensional cone architecture, design, and unidirectional entry mechanism to capture mosquitoes

**Fig. 2:**
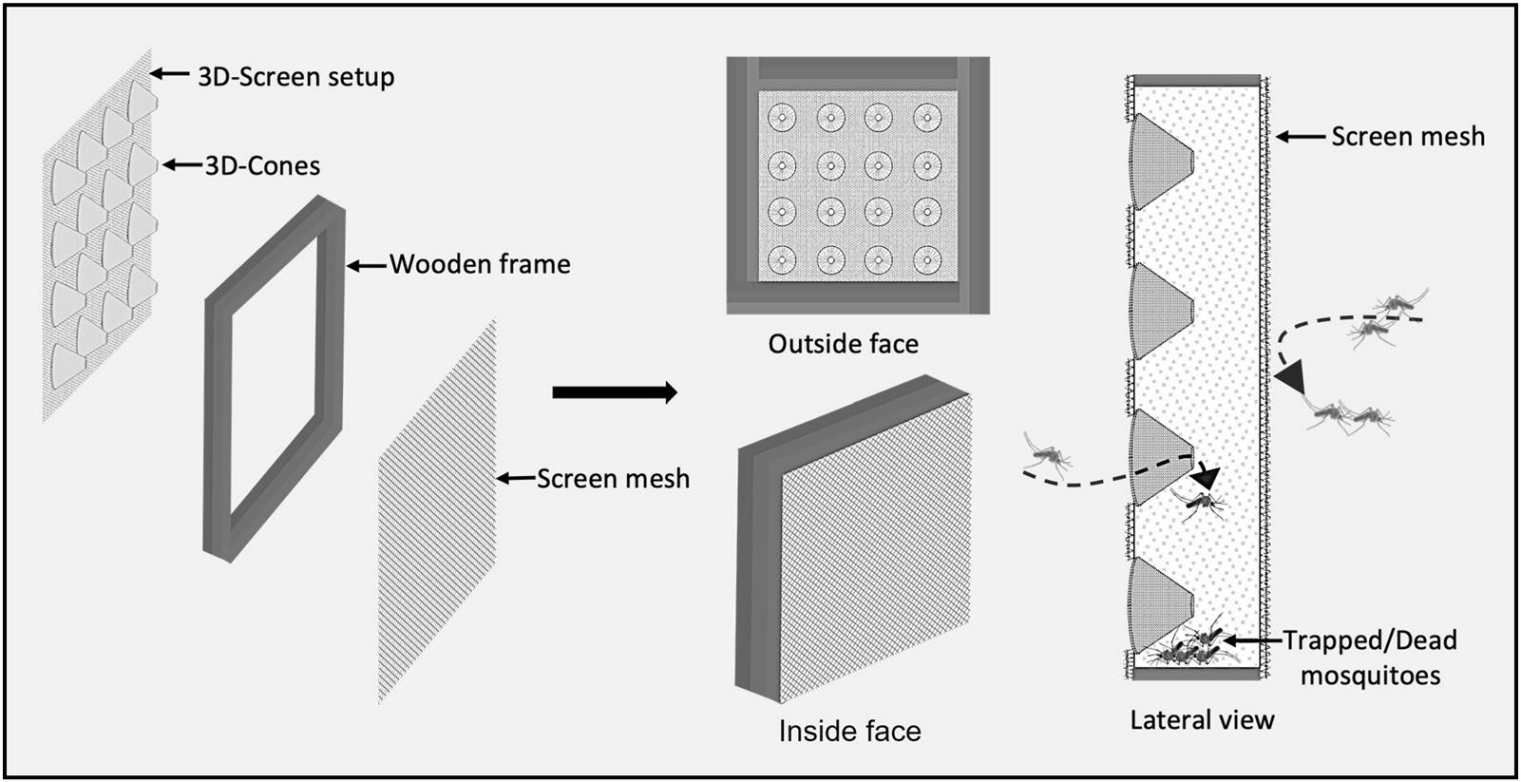
A 3D-Screen on one side (facing outside) and a traditional screen on the other side (facing inside) to form a 3D-WDST with a single 3D-Screen

**Fig. 3:**
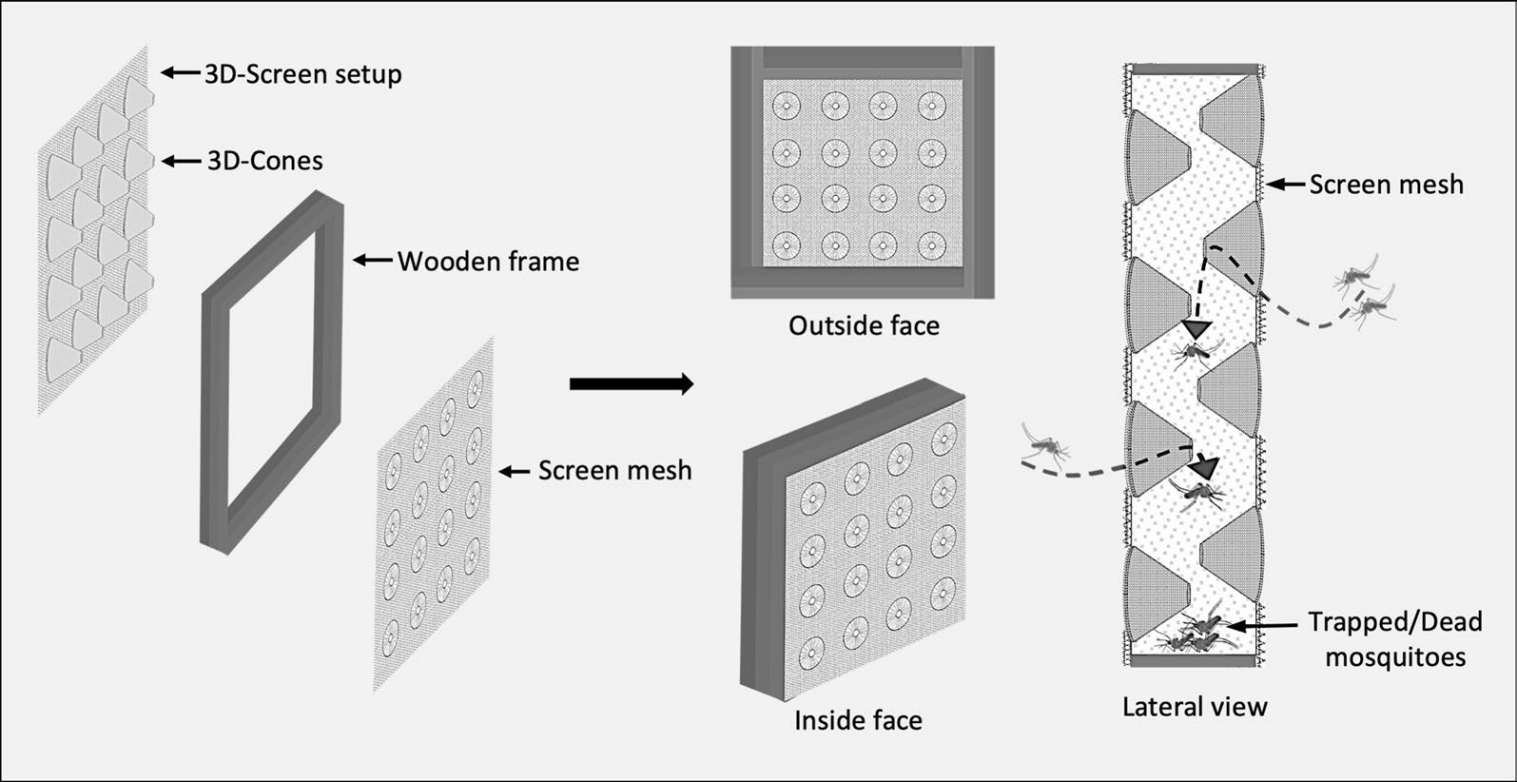
Three-dimensional screens on both sides (facing outside and inside) to form 3D-WDST with two 3D screens

The 3D-Screens were made of conical structures fitted on traditional screen mesh. The tip of each cone had a perforation to create a pore with a 5-mm diameter. The base of the cone was fully open, creating a hole with 5-cm diameter. The density of the cones on the screen mesh was 100 cones/m^2^ (Fig. 1). In a 3D-WDST setup, the larger opening of the cone faced outside of the window while the smaller tip faced inside allowing the free-flying mosquitoes to penetrate the screens from outside only but not the other side and get trapped in the space between the double-screen setups. 3D-Screens were mounted on a wooden frame, corresponding to the size of the window of experimental huts, using Velcro tape forming a 3D-WDST with either a 3D-Screen and a traditional screen mesh or with two 3D-Screens on both sides of the window frame (Figs. 2, 3). The study used two different cone types; one was made by hand from traditional window screen material (glass fiber-reinforced polyester screen purchased from a local hardware store) and the other from plastic by injection molding by Gidetec Oy, Pirkkala, Finland. Installation of the 3D-WDST on the experimental huts’ windows was conducted by local carpenters from the Muheza under the supervision of personnel from the University of Helsinki, Helsinki, Finland, and National Institute for Medical Research, Amani Medical Research Centre, Muheza, Tanzania.

### The study area and experimental huts

**Fig. 4:**
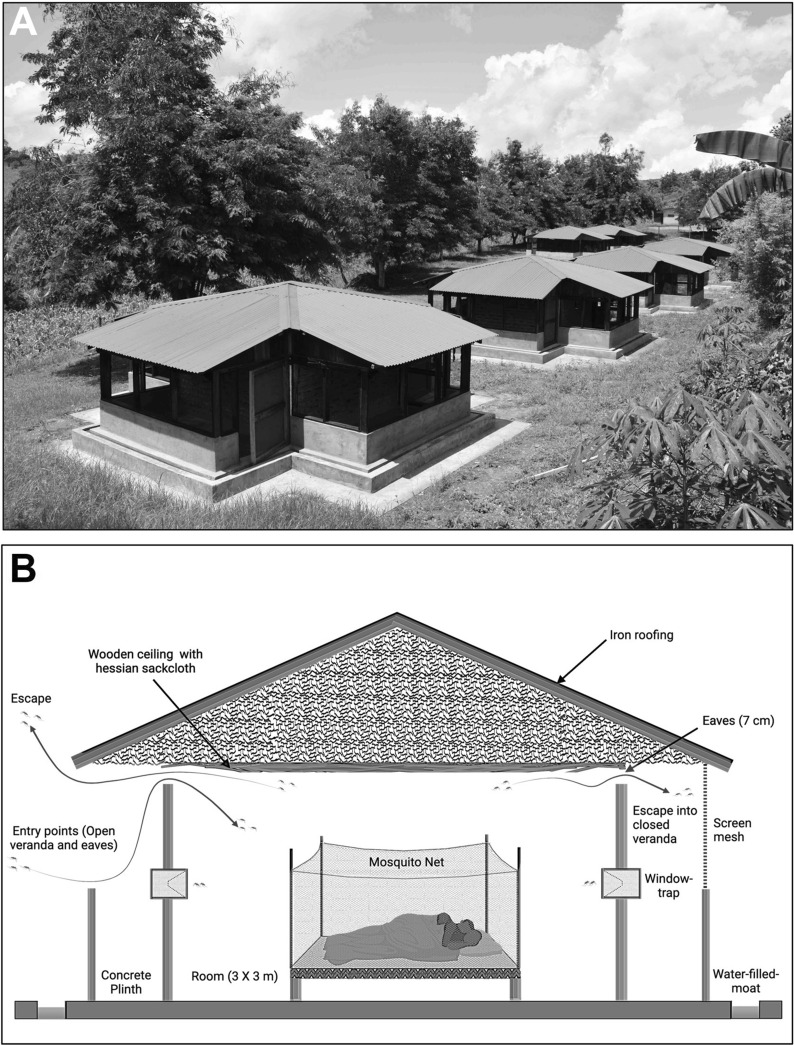
East African experimental huts. **A** Six experimental huts in Muheza, northeastern Tanzania. **B** Experimental hut design showing overall architecture of the hut, mosquito entry and exit point, and placement of window traps (exit traps are shown)

**Fig. 5:**
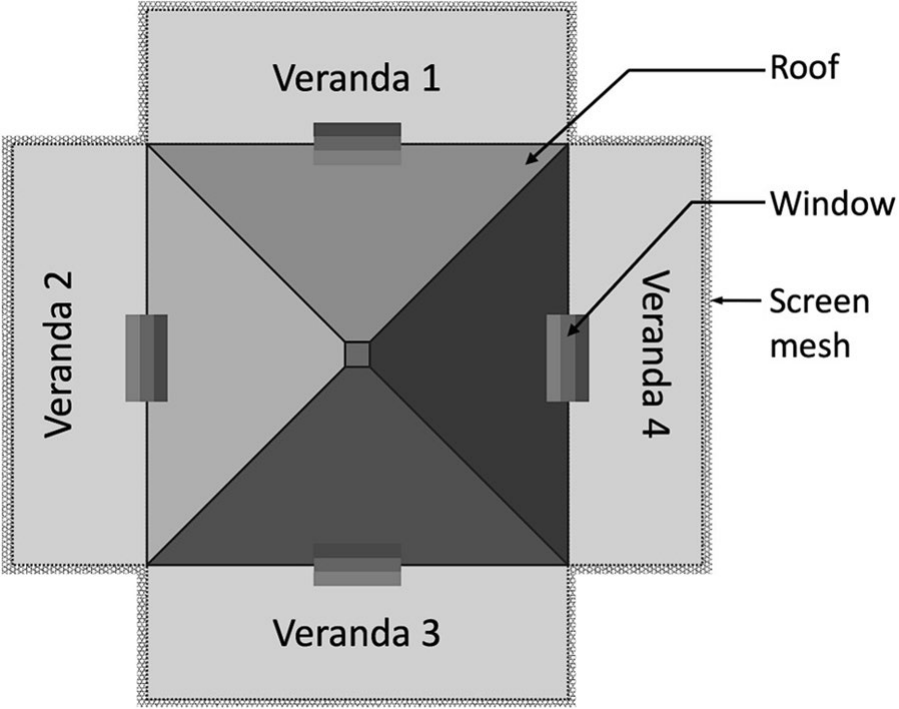
Top view of experimental hut showing the four verandas

This study was conducted in the Muheza district of the Tanga region in northeastern Tanzania. The experimental huts used in this study (Fig. 4A) are located at the National Institute for Medical Research field station at Zeneti village in Muheza district (5° 13′ S latitude, 38° 39′ E longitude and 193 m altitude) where *An. gambiae* s.l. is the predominant vector in the long rainy season while *An. funestus* s.l. is the main vector during the short rainy season [15, 16]. Experimental huts were constructed according to the design recommended by WHO and based on the original veranda-hut model developed in Tanzania in 1960s [17–19]. The huts are built on concrete plinths and surrounded by a water-filled moat to deter entry of scavenging ants (Fig. 4B). The huts are identical, made in a traditional design with brick walls plastered with mud on the inside, a wooden ceiling lined with hessian sackcloth, iron roof, open eaves, and a window on each side (4, including the window opening on the door). Every hut has four verandas, one on each side of the hut (Fig. 5). Verandas are not open to each other and can be screened or left open depending on the study design; for example, two opposite sides of the hut could have standard exit traps and screened verandas to capture mosquitoes leaving via the exit trap and the eaves (by collecting mosquito from exit trap and the veranda space). The other two sides could be left open (unscreened) so that mosquitoes could enter the hut through the eaves. We used five experimental huts during Trial I and six huts during Trial II and Trial III.

### Experimental hut study designs

**Fig. 6:**
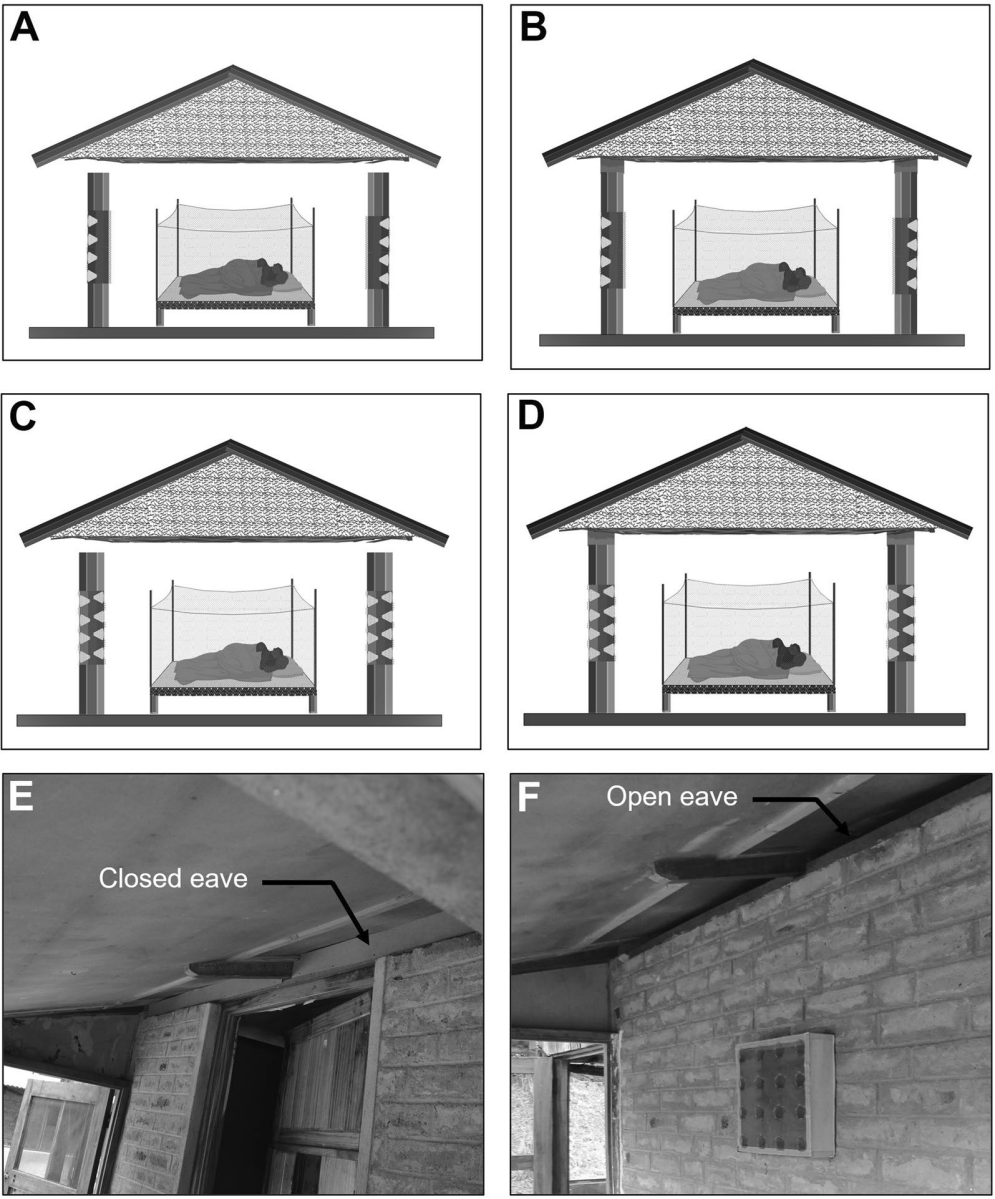
3D-Screen installation in experimental huts with open and closed eave configurations. **A** 3D-WDST with a single 3D-Screen and open eaves, **B** 3D-WDST with a single 3D-Screen and closed eaves, **C** 3D-WDST with two 3D-Screens and open eaves, **D** 3D-WDST with two 3D-Screens and closed eaves. **E** An example of closed eaves in one of the experimental huts, **F** an example of open eaves with 3D-WDST fixed on an experimental hut window

**Fig. 7:**
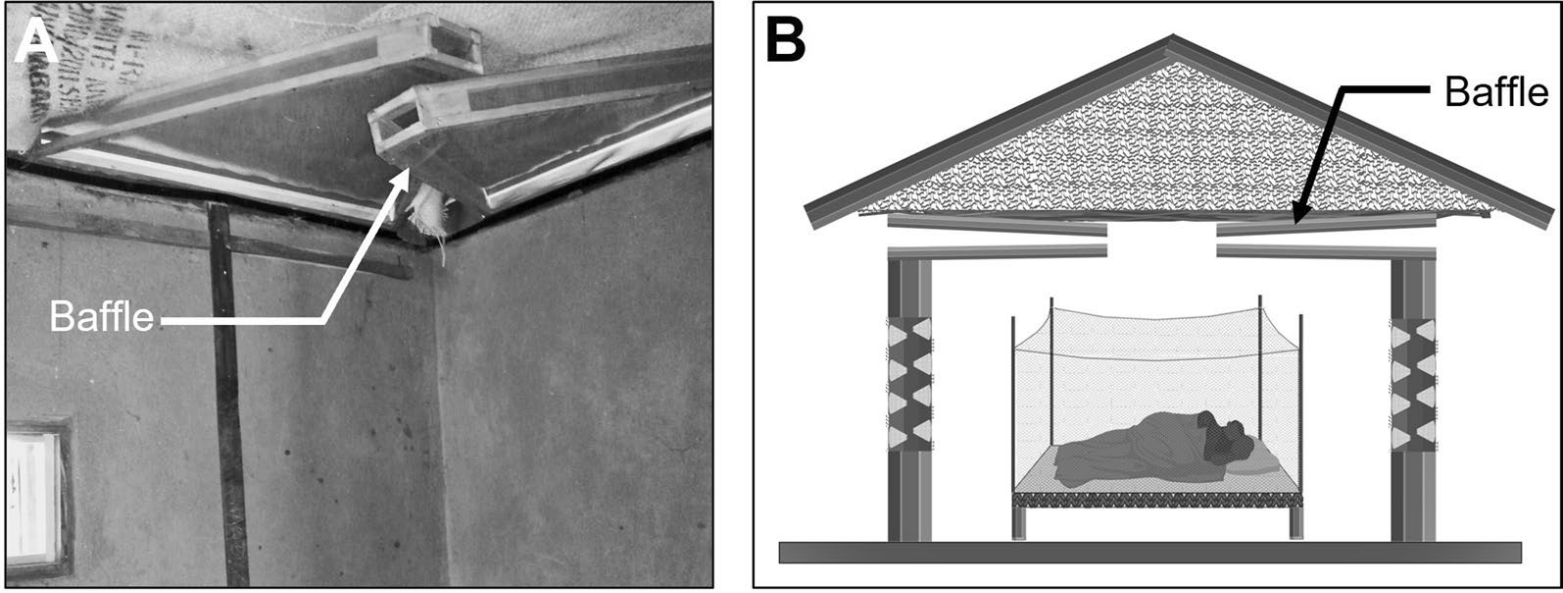
3D-WDST with two 3D-Screens installed on experimental huts with baffles. **A** 3D-WDST with baffles included, **B** baffles setup in one of the experimental huts

We conducted three experimental hut trials, Trial I (May–June 2016), Trial II (May–June 2017), and Trial III (November–December 2017), during which two versions of the 3D-WDST setup, a 3D-WDST made of a 3D-Screen and a traditional screen or two 3D-Screens (the tips of the cones were facing the inner space of the 3D-WDST) and two versions of the cone material (cones made of traditional screen, handmade, and made from plastic by injection molding, machine-made), were evaluated. The huts with fitted 3D-WDST were assigned as treatment huts, and the mosquito-capturing performance of the 3D-WDST was estimated and compared to a control hut. The control hut was fitted with the standard exit traps [22] on all windows to serve as a configuration that allows maximum capturing of host-seeking mosquitoes using a standard research mosquito exit trap. Figures 2 and 3 illustrate the configuration of using single and double 3D-Screens in 3D-WDST setup. The details of treatment arms in each trial are outlined below and summarized in Table 1. Installation of 3D-WDST in different hut condition is illustrated in Figs. 6 and 7.

**Table 1:** Summary of study arms and hut condition in three experimental hut trial

Trial no.	Trial date	Hut ID	Treatment code	Hut type	3D cone material	3D-WDST type	Eaves condition	Baffles condition	Veranda condition
1	May–June 2016	A	SO	Treatment	Mesh	Single	Open		All open
		B	SC	Treatment	Mesh	Single	Closed		All open
		C	DO	Treatment	Mesh	Double	Open		All open
		D	DC	Treatment	Mesh	Double	Closed		All open
		E	C	Control			Open		North and south open
2	May–June 2017	A	*C*_open_	Control 1				Removed	North and south open
		B	*T*_baffles_	Treatment	Mesh	Double		Included	All open
		C	*T*_open_	Treatment	Mesh	Double		Removed	All open
		D	*T*_baffles_	Treatment	Mesh	Double		Included	All open
		E	*C*_baffles_	Control 2				Included	All open
		F	*T*_open_	Treatment	Mesh	Double		Removed	All open
3	Nov–Dec 2017	A	*C*_open_	Control 2				Removed	All open
		B	HM_open_	Treatment	Mesh	Double		Removed	All open
		C	MM_baffles_	Treatment	Plastic	Double		Included	All open
		D	HM_baffles_	Treatment	Mesh	Double		Included	All open
		E	MM_open_	Treatment	Plastic	Double		Removed	All open
		F	*C*_baffles_	Control 1				Included	North and south open

### Trial I

The trial ran for 30 nights, and the following evaluation arms were studied:

i. SO–The 3D-WDST was made of a 3D-Screen facing the outside and a traditional screen facing the inside of the hut and the eaves were open.
ii. SC–The 3D-WDST was made of a 3D-Screen facing the outside and traditional screen facing the inside of the hut and the eaves were closed.
iii. DO–The 3D-WDST was made of two 3D-Screens on both sides of the double-screen setup and the eaves were open.
iv. DC–The 3D-WDST was made of two 3D-Screens on both sides of the double-screen setup and the eaves were closed.
v. C–The control hut was with exit traps on all four sides, north and south verandas and the eaves were open.

### Trial II

The trial ran for 36 nights, and the following evaluation arms were studied:

i. *T*_open_–The 3D-WDST was made of two 3D-Screens on both sides of the double-screen setup and the eaves were open**.**
ii. *T*_baffles_–The 3D-WDST was made of two 3D-Screens on both sides of the double-screen setup and the eaves had baffles.
iii. *C*_open_–The control hut was with exit traps on all four sides and all the verandas, and the eaves were open.
iv. *C*_baffles_–The control hut was with exit traps on all four sides, the north and south verandas were screened, and the eaves had baffles.

Six huts were used for the trial, and the treatments *T*_open_ and *T*_baffles_ were run in duplicates. Data analyses were performed from the average outcomes of each replicate of *T*_open_ and *T*_baffles_.

### Trial III

The trial ran for 36 nights, and the following evaluation arms were studied:

i. HM_open_–The cones of the 3D-Screens were made of traditional screen material, the 3D-WDST was made of two 3D-Screens on both sides of the double-screen setup, and the eaves were open.
ii. HM_baffles_–The cones of the 3D-Screens were made of traditional screen material, the 3D-WDST was made of two 3D-Screens on both sides of the double-screen setup, and the eaves had baffles.
iii. MM_open_–The cones of the 3D-Screens were manufactured from plastic, the 3D-WDST was made of two 3D-Screens on both sides of the double-screen setup, and the eaves were open.
iv. MM_baffles_–The cones of the 3D-Screens were manufactured from plastic, the 3D-WDST was made of two 3D-Screens on both sides of the double-screen setup, and the eaves had baffles.
v. *C*_baffles_–The control hut was with exit traps on all four sides, north and south verandas were screened, and the eaves had baffles.
vi. *C*_open_–The control hut had exit traps on all four sides, all verandas and the eaves were open.

### Hut procedure

**Fig. 8:**
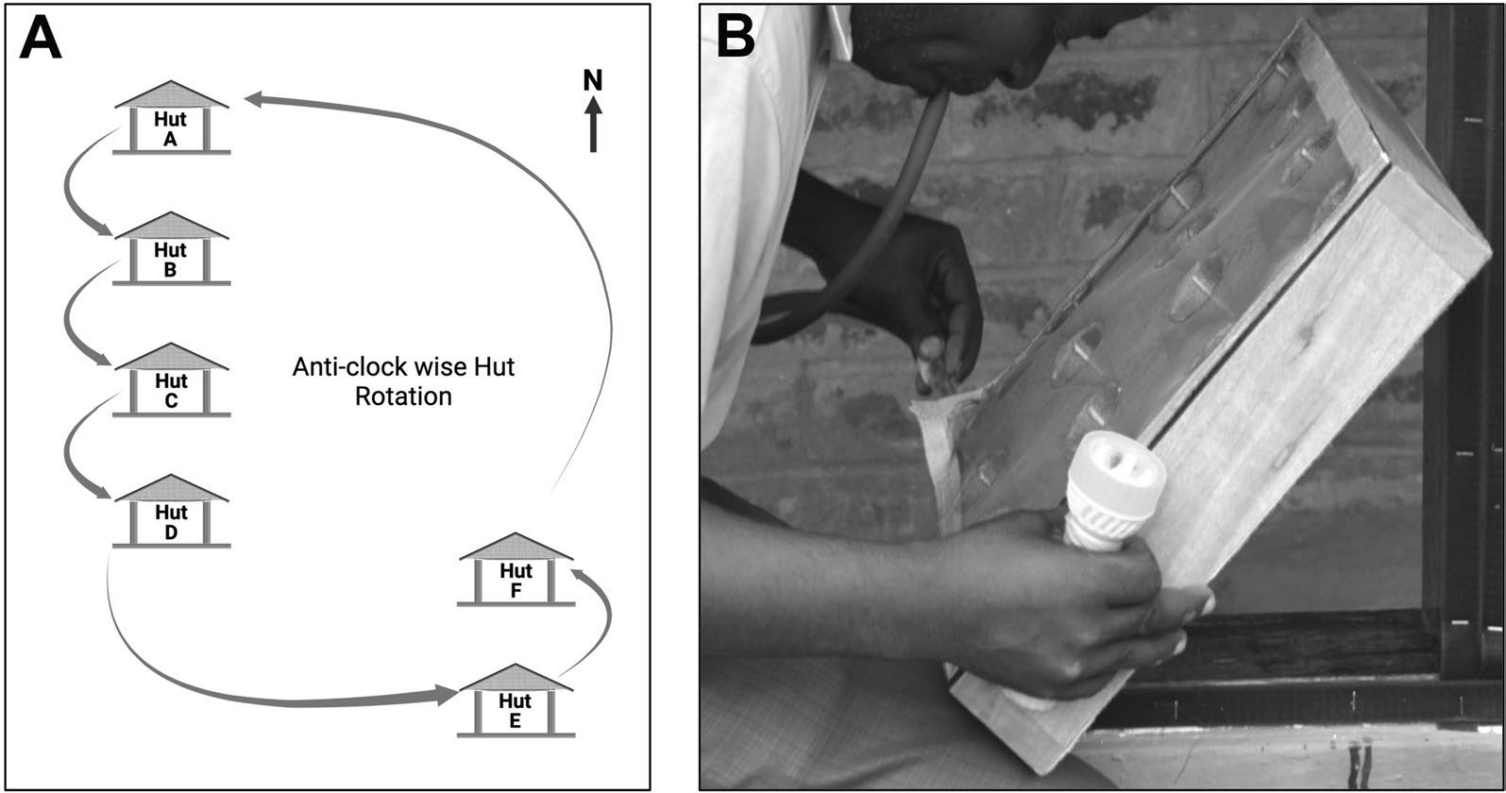
**A** Weekly hut rotation plan. **B** Field worker collecting trapped mosquitoes from the 3D-WDST

Two volunteers were recruited for each hut to sleep under untreated bed nets every test night from 1900 to 0630 h. Sleepers were alternated between the experimental huts on successive nights to remove possible bias due to differences in individual attractiveness to mosquitoes, and treatments were rotated weekly on the experimental huts to adjust for any differences in positional attractiveness of the huts using a Latin square design (Fig. 8A). Sundays of every week were reserved for introducing modification into the huts for the successive treatment allocation.

### Mosquito collection and storage

Mosquitoes were collected by a team of trained field entomologists every morning (except Sundays) from the 3D-WDSTs, the exit traps (control huts), and verandas (control huts). The 3D-Screens were affixed to the window frames using Velcro tapes. Mosquitoes trapped in the 3D-WDSTs were collected by inserting a hand-held mouth aspirator through a small opening created by temporarily detaching the Velcro tape (Fig. 8B). Hand-held mouth aspirators were also used to collect mosquitoes from the other compartments. Mosquitoes were also collected from inside the hut for 30 man-min. All live and dead mosquitoes were collected, sorted by gender, counted, and grouped in the field into anophelines and culicines based on morphological characteristics [20–22]. Grouped mosquitoes were transferred into paper cups, covered with mesh material, provided with cotton wool soaked in 10% glucose solution, and brought to the laboratory for further processing. In the laboratory, mosquitoes were identified to species using standard morphological keys. All identified female anophelines were stored over silica gel for further molecular analyses.

### Molecular analyses

Genomic DNA was extracted from whole-body samples of all female anopheline mosquitoes collected throughout the study. Mosquito specimens were transferred into 2-ml screw cap tubes containing 250 mg zirconia powder and six 3-mm silica glass beads, and a repeating bead-beating (RBB) was performed at 1400 rpm for 60 s (FastPrep-96™, MP Biomedicals, Irvine, CA). To the resulting homogenate, 200 µl lysis buffer (1 M Tris–HCL pH 8.0, 0.5 M EDTA, 5 M NaCl, and 20% SDS) was added, and RBB was performed three times with incubation at room temperature for 5 min after each step. Final homogenate was centrifuged at 9000*g* for 5 min, and the supernatant was transferred to a fresh 96-well plate. DNA extraction was conducted using the Kingfisher Flex Magnetic Particle Processor (ThermoFisher scientific) following manufacturer’s instructions. Quality control for the extracted DNA was performed using Quant-iT™ PicoGreen™ assay kit (ThermoFisher Scientific). DNA was finally stored in 96-well plates at − 20 °C for further molecular analyses.

Species identified as *An. gambiae* s.l. morphologically in the laboratory were further identified to the sibling species using polymeric chain reaction (PCR) method described previously [23]. To determine infection rate of the *Anopheles* mosquitoes with the malaria parasite, all mosquito DNA samples were subjected to *P. falciparum* infection analysis using nested PCR method as described by Snounou et al. [24]. PCR product size analysis was performed using automated capillary electrophoresis separation on the LabChip GX Touch HT Nucleic Acid Analyzer (Perkin Elmer) following the manufacturer’s instructions.

### Ethical consideration

This study was conducted following the guidelines of the Declaration of Helsinki and the international guidelines for ethical review of epidemiological studies. Both verbal and written consents were taken from the volunteers (sleepers) after explaining the nature of the study. The risk of malaria was explained, and all volunteers were provided chemoprophylaxis during the trial period and 1 month post trial period. All volunteers were also monitored daily for fever and malaria symptoms. All procedures for data collection, management, storage, and analysis followed standard operating procedures. Ethical clearance was sought from the ethics committees of the National Institute of Medical Research (NIMR) Tanzania (Ref: NIMR/HQ/R.8a/Vol. IX/2399).

### Data analyses

Raw data were collected in the field using a daily record sheet prepared and customized for each trial. Recordings were further entered into an Excel spreadsheet (Microsoft Excel 2015, Microsoft®, New York, USA) the following day. Mosquito specimens were recorded as the number of daily catches and species from traps, rooms, and verandas. Mosquito recording was further summarized, adjusted, and represented as follows.

- Total collection from 3D-WDST (*T*_*T*_) = sum of collection from the 3D-WDSTs from east, west, north, and south wing.
- Total collection from exit traps (*T*_ET_) = sum of collection from the exit traps from the control huts from east, west, north, and south wing.
- Catches from room, treatment hut, (*T*_RT_) = total collection from room obtained after 30 man-min of manual catching using a mosquito aspirator.
- Catches from room, control hut, (*T*_RC_) = total collection from room obtained after 30 man-min of manual catching using a mosquito aspirator.
- Total collection in treatment huts (*T*_TR_) = *T*_T_ + *T*_RT_
- Total collection in control huts (*T*_C_) = (2 × Verandas) + *T*_ET_ + *T*_RC_

The total numbers of mosquitoes in the two veranda traps from the control hut were multiplied by two to adjust for the unrecorded escapes through the other two verandas which were left unscreened to allow routes for entry of mosquitoes via the eaves [25, 26].

Two alternative methods were used to calculate the efficacy of the 3D-WDST in capturing mosquitoes entering the treatment huts. The first calculated the capturing efficacy percentage relative to the total number of mosquitoes collected from both the 3D-WDSTs and the room of the respective treatment. On the other hand, the second calculated the capturing efficacy percentage relative to the total number of mosquitoes collected from both the exit traps and the room of the control hut.

$$\mathrm{Trapping\;efficacy\;relative\;to\;the\;total\;mosquitoes\;collected\;from\;the\;treatment\;hut}\left({E}_{T}\%\right)= \frac{{T}_{T}}{{T}_{TR}} \times 100\%$$

$$\mathrm{Trapping\;efficacy\;relative\;to\;the\;total\;mosquitoes \;collected\;from\;the\;control\;hut }\left({E}_{c}\%\right)= \frac{{T}_{T}}{{T}_{c}} \times 100\%$$

Excel data were further summarized and converted into pivot tables to summarize the collection in different setup regarding species, collection hut, and gonotrophic statuses of specimen.

## Statistical analysis

Data are presented as means and standard deviations (SD). Shapiro-Wilk test for normality was performed. For data sets returning normal distribution, *t* test was used when comparing two groups, and one-way analysis of variance (ANOVA) followed by post hoc Tukey’s multiple pairwise comparisons or Fisher's least significant difference (LSD) test was used when comparing more than two groups. A probability value of *P* < 0.05 was indicative of statistical significance in all tests. All calculations were performed using the GraphPad Prism 9 software package (GraphPad Software, San Diego, CA, USA). Descriptive statistics and statistical analyses to establish difference between treatment arms and plots generation were also conducted using GraphPad Prism.

## Results

**Fig. 9:**
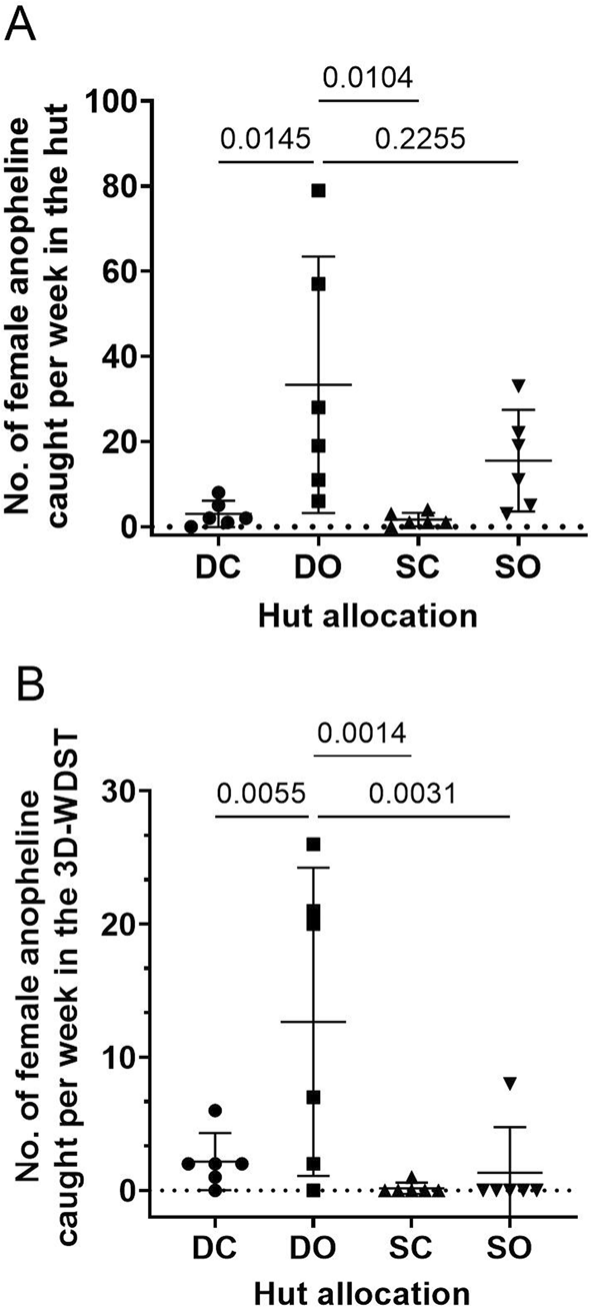
Weekly anopheline count in each treatment (each point represents mean mosquito number collected during the 6-week collection period). **A** Weekly mean anopheline collection from the treatment huts. **B** Weekly mean anopheline collection from the 3D-WDST from each treatment hut. DC: double 3D-Screen and closed eaves, DO: double 3D-Screens and open eaves, SC: single 3D-Screen and closed eaves, SO: single 3D-Screen and open eaves

**Fig. 10:**
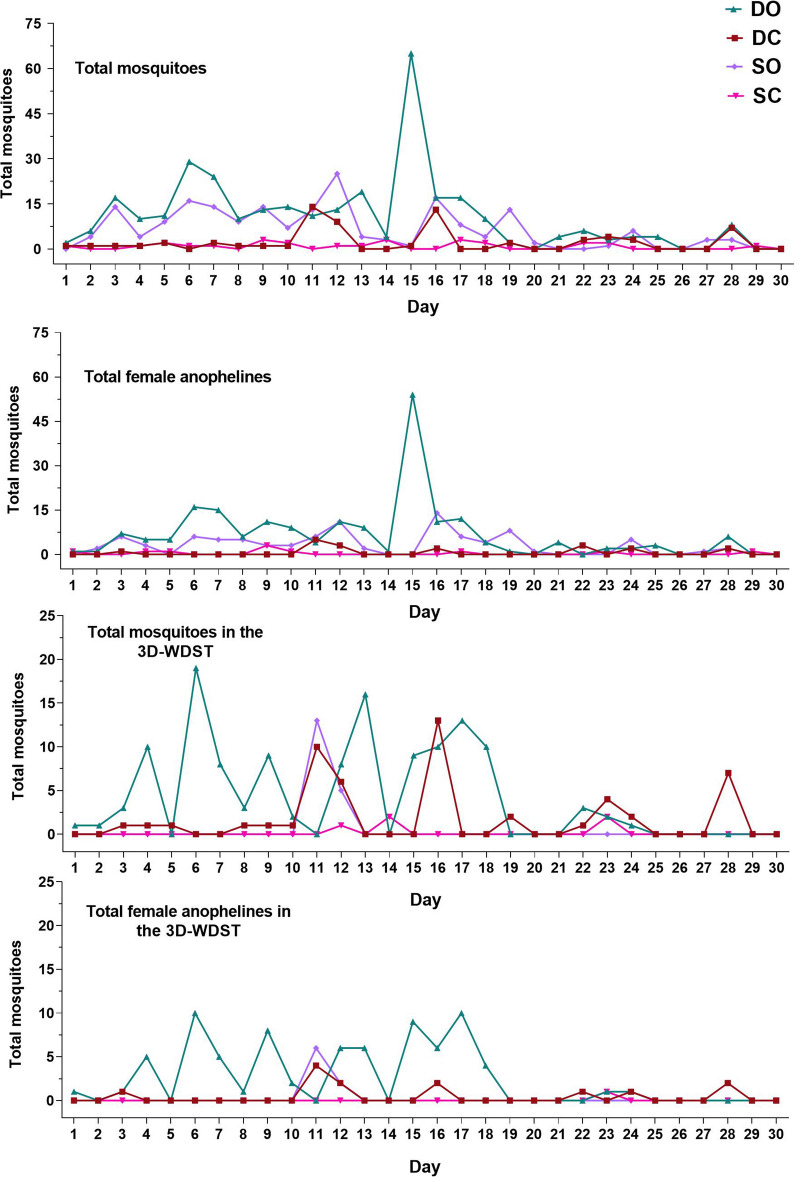
Nightly mosquito counts for different treatment conditions during 30-day collection period. DC: double 3D-Screen and closed eaves, DO: double 3D-Screens and open eaves, SC: single 3D-Screen and closed eaves, SO: single 3D-Screen and open eaves

In Trial I, we investigated whether a 3D-WDST with a single 3D-Screen (SO and SC treatments) or with two 3D-Screens (DO and DC treatments) would perform better in capturing mosquitoes under semi-field conditions. In the same experimental setup, we also investigated whether closing the eaves of the huts (SC and DC treatments) or leaving them open (SO and DO treatments) would affect the mosquito-capturing capacity of the 3D-WDST. Results of these comparisons, including the average weekly (5 days) mosquito counts, statistical parameters, and significant differences are summarized in Table 2 and presented graphically in Fig. 9A, B. The nightly mosquito counts within each treatment are also shown in Fig. 10.

**Table 2:** Mean mosquito counts per week in different test conditions

	DC	DO	SC	SO
	Mean (95% CI)	Mean (95% CI)	Mean (95% CI)	Mean (95% CI)
Mosquitoes in the Huts				
Total specimen	11.17 (3.56–18.78)	53.83 (11.87–95.79)	4.33 (2.27–6.39)	32.33 (9.22–55.45)
Female	8.50 (1.93–15.06)	45.17 (6.69–83.64)	3.00 (0.61–5.39)	22.5 (5.84–39.16)
Anophelines	4.67 (0.81–8.51)	40.67 (7.60–73.74)	3.00 (1.67–4.32)	24.33 (7.53–41.13)
Female anophelines	3.60 (0.02–7.17)	33.33 (3.19–63.48)	2.00 (0.244–3.75)	15.5 (3.55–27.44)
Culicines	6.50 (1.82–11.18)	13.17 (3.42–22.91)	1.33 (*− *0.10–2.767)	8 (0.94–15.06)
Female culicines	5.50 (1.47–9.524)	11.83 (2.74–20.92)	1.33 (*− *0.10–2.76)	7 (0.46–13.54)
Mosquitoes in 3D-WDST only				
Total specimen	8.50 (2.5–14.5)	21.33 (3.84–38.83)	0.83 (*− *0.56–2.22)	3.00 (*− *4.71–10.71)
Female	6.00 (1.6–10.4)	17.50 (2.61–32.38)	0.33 (*− *0.20–0.87)	1.67 (*− *2.61–5.95)
Anophelines	3.83 (0.42–7.24)	15.67 (1.6–29.74)	0.67 (*− *0.417–1.75)	2.67 (*− *4.18–9.52)
Female anophelines	2.16 (0.03–4.31)	12.67 (1.10–24.23)	0.17 (*− *0.26–0.60)	1.33 (*− *2.1–4.76)
Culicines	4.67 (1.23–8.1)	5.67 (1.13–10.2)	0.17 (*− *0.26–0.60)	0.33 (*− *0.52–1.19)
Female culicines	3.83 (1.06–6.60)	4.83 (0.77–8.90)	0.17 (*− *0.26–0.60)	0.33 (*− *0.52–1.19)

A total of 996 mosquitoes were collected during the collection period of 30 nights of which 752 were morphologically identified as anophelines (28.05% male, *n* = 211 and 71.94% female, *n* = 541) and 244 as culicines (14.75% male, *n* = 36 and 85.24% female, *n* = 208). Further morphological identification of anophelines identified 433 mosquito specimens as *An. gambiae* s.l. (30.02% male, *n* = 130 and 69.97% female, *n* = 303) and 319 specimens as *An. funestus* s.l. (25.39% male, *n* = 81 and 74.60% female, *n* = 238). Female anophelines were sorted and analyzed for *P. falciparum* infection analysis. Female anophelines morphologically identified as *An. gambiae* s.l. were subjected to PCR assay for sibling species identification (*n* = 303). From the molecular analyses, 288 were *An. gambiae* s.s. and 15 were *An. arabensis*. Additionally, 37 specimens were found to be infected with *P. falciparum*, 15 were *An. gambiae* s.s., and 22 were *An. funestus* s.l., while none were *An. arabiensis*. In the 3D-WDST of the DO hut condition, 1/48 and 1/28 captured *An. gambiae* s.s. and *An. funestus* s.l. were *P. falciparum* positive, respectively. In the 3D-WDST of the SO hut condition, 0/6 and 1/2 captured *An. gambiae* s.s. and *An. funestus* s.l. were *P. falciparum* positive, respectively. The other two treatment conditions, DC and SC, had no *P. falciparum*-infected mosquitoes in the 3D-WDST.

The ANOVA test provided strong evidence of a significant difference (*P* = 0.0048) between the weekly mean female anopheline mosquito counts inside the 3D-WDST of at least one pair of the four treatments. Tukey's multiple comparison test was carried out for all possible pairs of treatments (6 pairs). There was strong evidence (*P* = 0.0055, *P* = 0.0014, and *P* = 0.0031, adjusted *P* value) of a significant difference between DO treatment and DC, SC, and SO treatment (Fig. 9B), respectively. There was no evidence of a significant difference between the other pairs. The weekly mean female anopheline mosquito count in the 3D-WDST for the DO treatment was 12.67 (CI 1.10–24.23) compared to 2.16 (CI 0.025–4.31), 0.17 (CI − 0.261–0.60), and 1.33 (Cl − 2.1–4.76) for the DC, SC, and SO, respectively. The mosquito capturing efficacy of the 3D-WDST setup in the DO treatment was 33.11% (CI 7.40–58.81) relative to the total number of female anopheline mosquitoes collected in the DO treatment hut and 27.27% (CI 4.23–50.31) relative to the female anopheline mosquitoes collected in the control hut. Altogether, 3D-WDST built with two 3D-Screens (the permissive sides of the 3D-Screens were facing the outside and the inside of the hut) in combination with leaving the hut eaves open, i.e. the DO treatment, captured more mosquitoes than the rest of the experimental designs, i.e. the DC, SC, and SO treatments (Table 3). Therefore, the DO setup was used in a second trial to investigate the effect of using eave baffles that prevent mosquito escape through the eaves on the capturing efficacy of the 3D-WDST.

**Table 3:** Capturing efficacy of the 3D-WDST in the open eave setup

	DO capturing efficacy relative to the treatment hut	DO capturing efficacy relative to control hut
	% (95% CI)	% (95% CI)
Total mosquitoes	34.66 (10–59.33)	26.79 (7.18–46.39)
Total Female	34.57 (8.25–60.9)	30.47 (6.98–53.96)
Anophelines		
Total	32.43 (8.85–56.01)	23.77 (5.16–42.39)
Female	33.11 (7.40–58.81)	27.27 (4.23–50.31)
Culicines		
Total	39.59 (10.07–69.1)	48.25 (9.05–87.46)
Female	37.59 (8.66–66.53)	67.37 (-7.43–142.2)

Trial I demonstrated that 3D-WDST fitted to windows of experimental huts with open eaves captured significantly more mosquitoes than those fitted to huts with closed eaves. This suggests that a significant number of mosquitoes entered the huts through the eaves and got captured in the 3D-WDST while leaving the hut space in the early morning. Still another route for mosquitoes leaving the hut space in the morning is the eaves themselves. Therefore, we asked what would the 3D-WDST capturing efficacy be if we permitted mosquito entry through the eaves and block mosquito escape through them using baffles? To answer this question, Trial II was conducted in which four huts were fitted with 3D-WDST; two of them had open eaves (*T*_open_) while the other two had baffles (*T*_baffles_) fixed in the eaves. Two more huts served as controls (one with open eaves and the other with baffles), and the trial ran for 6 weeks with weekly rotation of huts and daily rotation of sleepers.

**Fig. 11:**
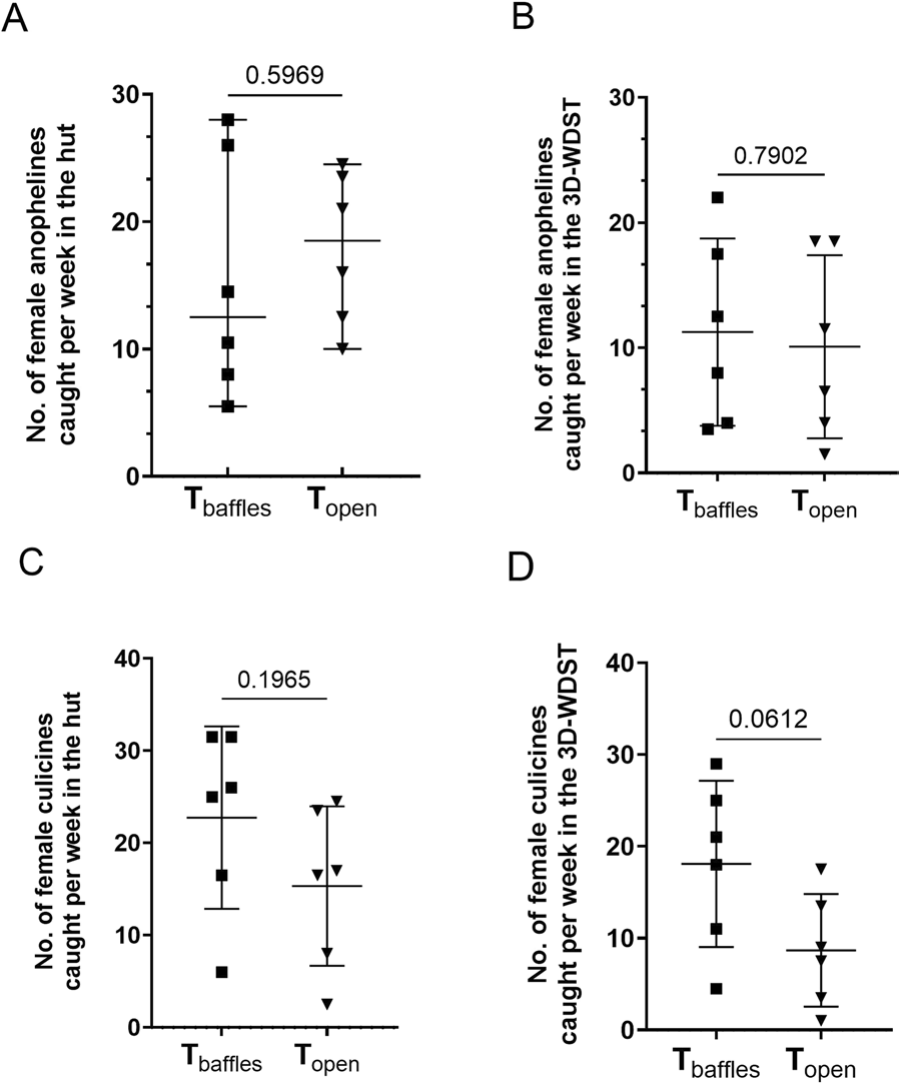
Weekly mosquito count in the huts with and without baffles (each point represents mean mosquito number collected during the 6-week collection period). **A** Weekly mean female anopheline collection from the huts with and without baffles. **B** Weekly mean female anopheline collection from the 3D-WDST from huts with and without baffles. **C** Weekly mean female culicine collection from the huts with and without baffles. **D** Weekly mean female culicine collection from the 3D-WDST from huts with and without baffles

**Fig. 12:**
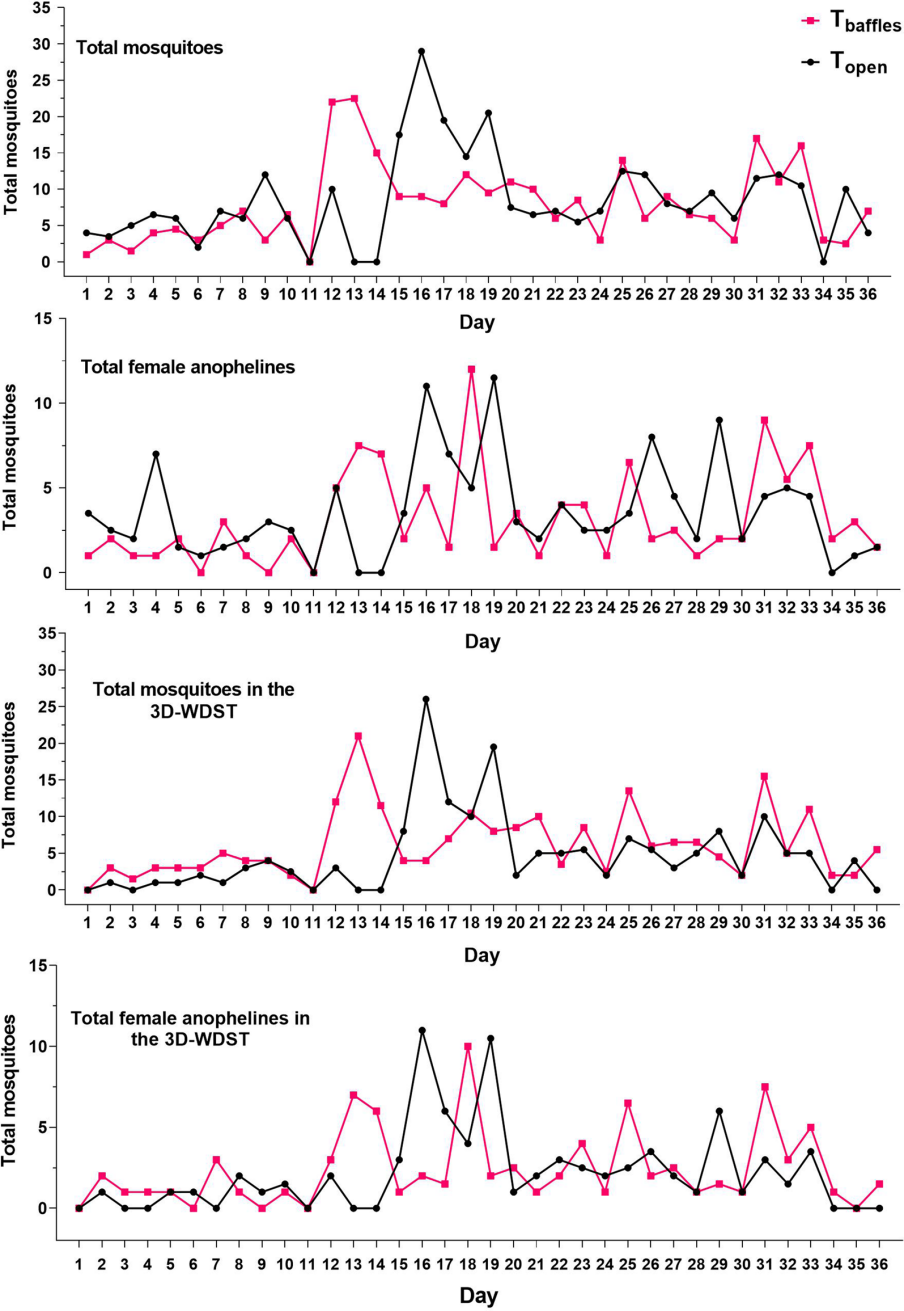
Nightly mosquito counts from *T*_open_ and *T*_baffles_ conditions during 36-day collection period

Data of this comparison, including the weekly (6 days) mean mosquito counts, statistical parameters, and significant differences, are summarized in Table 4 and presented graphically in Fig. 11. The nightly mosquito counts within each treatment are also shown in Fig. 12.

**Table 4:** Mean mosquito counts per week in different test conditions

	*T*_baffles_	*T*_open_
	Mean (95% CI)	Mean (95% CI)
Mosquitoes in the huts		
Total specimen	44.83 (24.56–65.1)	46.5 (30.86–62.14)
Female	38.17 (19.76–56.57)	33.25 (19.42–47.08)
Anophelines	21.00 (9.53–32.46)	29.08 (20.49–37.68)
Female anophelines	15.42 (5.47–25.36)	17.92 (11.63–24.21)
Culicines	23.83 (12.92–34.75)	17.42 (7.29–27.54)
Female culicines	22.75 (12.38–33.12)	15.33 (6.26–24.4)
Mosquitoes in 3D-WDST only		
Total specimen	33.58 (16.22–50.94)	22.25 (6.35–38.15)
Female	29.33 (13.86–44.81)	18.75 (4.87–32.63)
Anophelines	14.58 (5.19–23.98)	12.67 (2.93–22.4)
Female anophelines	11.25 (3.40–19.09)	10.08 (2.4–17.76)
Culicines	19.00 (9.20–28.81)	9.58 (3.17–16)
Female culicines	18.08 (8.57–27.59)	8.67 (2.22–15.11)

A total of 1729 mosquitoes were collected during the collection period of 36 nights of which 946 were anophelines (35.84% male, *n* = 339 and 64.16% female, *n* = 607) and 783 were culicines (7.66% male, *n* = 60 and 92.34% female, *n* = 723). Anophelines consisted of 42.50% *An. gambiae* s.l. (9.20% male, *n* = 37 and 90.70% female, *n* = 365) and 47.50% *An. funestus* s.l. (55.51% male, *n* = 302, and 44.48% female, *n* = 242). Of 365 female anophelines morphologically identified as *An. gambiae* s.l. that were subjected to PCR assays for sibling species identification, 360 were *An. gambiae* s.s. and 5 were *An. arabensis*. In addition, PCR assay for *P. falciparum* detection was performed on all female anophelines (*n* = 607) of which *P. falciparum* infection was found in 28 *An. gambiae* s.s. and 14 in *An. funestus* s.l. while none were found in *An. arabiensis*. In the 3D-WDST of the *T*_open_ hut condition, 8/97 and 1/24 captured *An. gambiae* s.s. and *An. funestus* s.l. were *P. falciparum* positive, respectively. In the 3D-WDST of the *T*_baffles_ hut condition, 5/73 and 2/62 captured *An. gambiae* s.s. and *An. funestus* s.l. were *P. falciparum* positive, respectively.

The *t*-test did not show a significant difference (*P* = 0.79) between the weekly mean female anopheline mosquito counts inside the 3D-WDST of *T*_open_ and *T*_baffles_. The weekly mean female anopheline mosquito count in the 3D-WDST of *T*_open_ was 10.08 (CI 2.4–17.76) and in the 3D-WDST of T_baffles_ was 11.25 (CI 3.40–19.09). The mosquito capturing efficacy relative to the total number of female anopheline mosquitoes collected in the respective treatment hut was 51.07% (CI 21.72–80.41) and 70.32% (CI 56.87–83.77) for *T*_open_ and *T*_baffles_, respectively. The mosquito-capturing efficacy relative to the total number of female anopheline mosquitoes collected in the respective control hut was 42.41% (CI 14.77–70.05) and 135.1% (CI 42.88–227.3) for *T*_open_ and *T*_baffles_, respectively. The capturing efficacy of 3D-WDST in hut conditions *T*_open_ and *T*_baffles_ is summarized in Table 5.

**Table 5:** Capturing efficacy of 3D-WDST setup in huts with baffles (T_baffles_) and without baffles (T_open_)

	Capturing efficacy relative to the treatment hut		Capturing efficacy relative to the control hut	
	*T*_baffles_ % (95% CI)	*T*_open_ % (95% CI)	*T*_bafles_ % (95% CI)	*T*_open_ % (95% CI)
Total mosquitoes	73.33 (61.79–84.88)	43.08 (19.87–66.29)	124.1 (48.33–199.9)	29.37 (13.59–45.14)
Female	75.04 (64.59–85.48)	50.41 (24.45–76.38)	121.1 (52.27–190)	34.75 (14.48–55.03)
Anophelines				
Total	67.93 (54.44–81.41)	39.97 (16–63.94)	131.7 (35.85–227.6)	31.23 (12.31–50.15)
Female	70.32 (56.87–83.77)	51.07 (21.72–80.41)	138.6 (37.23–239.9)	42.41 (14.77–70.05)
Culicines				
Total	78.14 (69.56–86.72)	52.91 (36.29–69.53)	135.1 (42.88–227.3)	30.65 (13.41–47.89)
Female	77.65 (68.5–86.79)	53.24 (33.78–72.7)	125.5 (46.03–205)	32.4 (11.71–53.09)

Interestingly, using baffles led to an increase in the number of female culicine mosquitoes in the 3D-WDST of the *T*_baffles_ compared with the *T*_open_, although the difference was not statistically significant (*P* = 0.06), Fig. 11D. The weekly mean female culicine mosquito count in the 3D-WDST of *T*_open_ was 8.67 (2.22–15.11) and in the 3D-WDST of *T*_baffles_ was 18.08 (CI 8.57–27.59).

In Trial III, we tested two production methods for the cone structures used to produce the 3D-Screens of the 3D-WDST. The cones that were used in Trial I and II were made of screen mesh material of the same type used to hold the cones and produce the setup of the 3D-WDST. These cones were handmade (HM), although laser was used to cut the mesh into the desired shapes. To improve integrity, reproducibility, and manufacturability of the cones, they were also produced in a factory using the injection molding manufacturing process (machine-made, MM). In Trial III, HM and MM cones were used to produce the 3D-Screen for the 3D-WDST setup. HM- and MM-based 3D-WDST were then tested under experimental hut conditions to compare their efficacy in mosquito capturing. Experimental huts with the baffle system (HM_baffles_ and MM_baffles_) were also included in the experimental design in addition to the open eave huts (HM_open_ and MM_open_) to replicate Trial II in the context of a different rainy season since Trial I and II were conducted in May–June 2016 and 2017 (long rainy season), respectively, while Trial III was conducted in November–December 2017 (short rainy season).

**Fig. 13:**
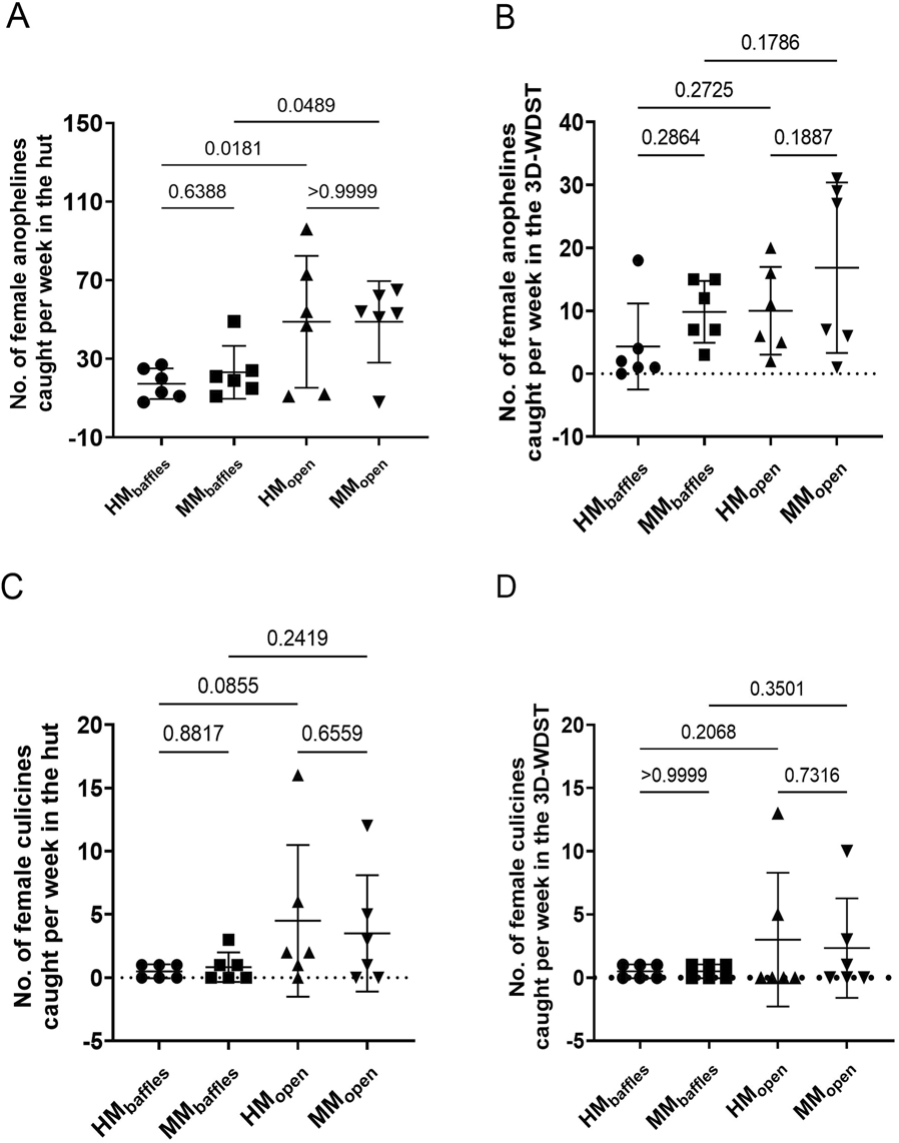
Weekly mosquito count in four different hut conditions (each point represents mean mosquito number collected during the 6-week collection period). **A** Weekly mean female anopheline collection from the treatment huts. **B** Weekly mean female anopheline collection from the 3D-WDST of each hut condition. **C** Weekly mean female culicine collection from the treatment huts. **D** Weekly mean female culicine collection from the 3D-WDST of each hut condition

**Fig. 14:**
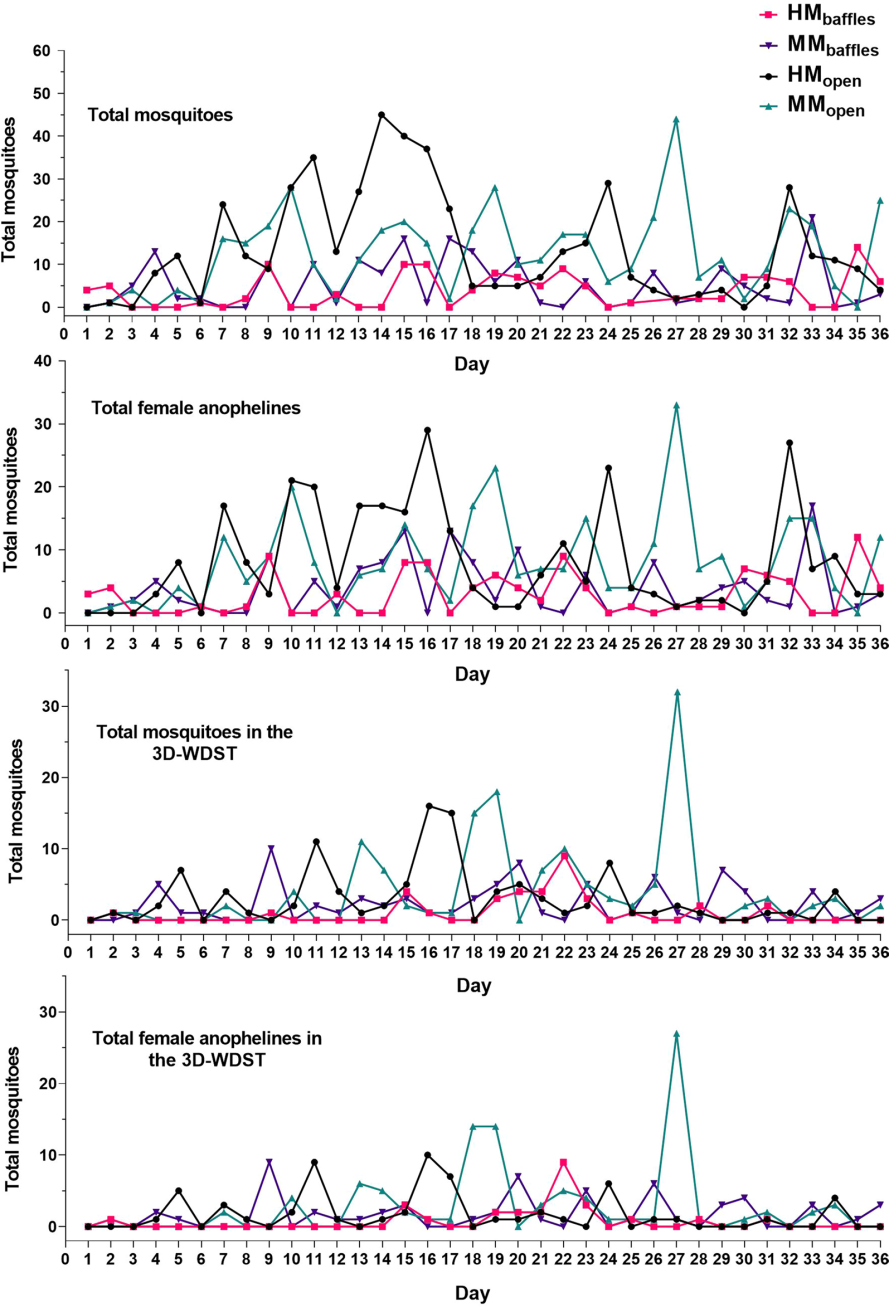
Nightly mosquito counts from different hut conditions during 36-day collection period

Data of the different hut conditions, including the weekly (6 days) mean mosquito counts, statistical parameters, and significant differences, are summarized in Table 6 and presented graphically in Fig. 13. The nightly mosquito counts within each treatment are also shown in Fig. 14. Of 2087 mosquitoes collected from a 36-day collection period during this trial, 1931 were anophelines (26 were *An. gambiae* s.l. and 1905 were *An. funestus* s.l.) and 156 were culicines. Since a significant proportion of anophelines were morphologically characterized as *An. funestus* s.l. (98.65%), we did not perform PCR assays for sibling species identification; however, a subset of daily female anophelines (57%, *n* = 772 of 1356) collected from each treatment regarding their collection wing were randomly selected for *Plasmodium* infection analysis. Of 772 mosquitoes selected for PCR assay, 22 were infected, and all infected specimens were *An. funestus* s.l. In the 3D-WDST of the MM_open_ and MM_baffles_ conditions, 1/54 and 1/58 tested *An. funestus* s.l. specimens (57% of total collected) were *P. falciparum* positive, respectively. The other two treatment conditions, HM_open_ and HM_baffles_, had no *P. falciparum*-infected mosquitoes in the 3D-WDST.

**Table 6:** Mean mosquito count per week in different test conditions

	HM_open_	MM_open_	HM_baffles_	MM_baffles_
	Mean (95% CI)	Mean (95% CI)	Mean (95% CI)	Mean (95% CI)
Mosquitoes in the huts				
Total specimen	80.5 (17.17–143.8)	74.67 (41.07–108.30)	21.7 (10.91–32.42)	31.2 (13.6–48.74)
Female	53.33 (13.47–93.2)	52.33 (27.95–76.72)	17.83 (9.27–26.4)	24 (9.46–38.54)
Anophelines	73.8 (16.12–131.5)	69.67 (38.28–101.1)	20.7 (10.56–30.77)	30 (12.92–47.08)
Female anophelines	48.8 (13.63–84.04)	48.83 (27.07–70.60)	17.33 (9.13–25.53)	23.2 (9.04–37.28)
Culicines	6.67 (− 0.40–13.74)	6 (− 0.01–12.020)	1.5 (− 0.55–3.55)	1.167 (− 0.06–2.40)
Female culicines	4.5 (− 1.79–10.79)	3.5 (− 1.32–8.321)	0.5 (− 0.07–1.08)	0.83 (− 0.40–2.06)
Mosquitoes in 3D-WDST only				
Total specimen	17.5 (3.75–31.24)	23.33 (3.11–43.55)	5.83 (− 3.13–14.80)	13.33 (8.16–18.5)
Female	13 (1.01–24.98)	19.17 (2.20–36.13)	4.83 (− 2.54–12.21)	10.33 (5.16–15.5)
Anophelines	12.67 (4.31–21.03)	20.17 (3.26–37.07)	6 (− 3.93–15.93)	12.5 (7.03–17.96)
Female anophelines	10 (2.70–17.3)	16.83 (2.62–31.04)	5.2 (− 3.81–14.21)	9.83 (4.67–15.00)
Culicines	4.83 (− 1.85–11.52)	3.16 (− 1.50–7.83)	0.83 (− 0.40–2.06)	0.83 (0.04–1.62)
Female culicines	3 (− 2.55–8.55)	2.33 (− 1.80–6.46)	0.5 (− 0.07–1.075)	0.5 (− 0.07–1.07)

The data showed that the weekly mean female anopheline mosquito count in the 3D-WDST of HM_open_ was 10 (2.70–17.3) and in the 3D-WDST of MM_open_ was 16.83 (2.62–31.04). Although there were more mosquitoes in the 3D-WDST of the MM_open_ condition than in the HM_open_ condition, 16.83 vs. 10, respectively, the uncorrected Fisher's LSD test did not show a significant difference (*P* = 0.1887) between the weekly mean female anopheline mosquito counts inside the 3D-WDST of the two conditions. However, these data suggest that the cones made by injection molding, i.e. the MM cones, showed a similar or even better performance to that of the cones made of screen mesh, i.e. the HM cones. These findings warrant production of durable cones at industrial scale for future large-scale studies to evaluate 3D-WDST at community levels. Further analyses of the data showed that the mosquito-capturing efficacy relative to the total number of female anopheline mosquitoes collected in the respective treatment hut was 24.44% (8.06–40.82) and 30.55% (9.386–51.71) for HM_open_ and MM_open_, respectively. The mosquito capturing efficacy relative to the total number of female anopheline mosquitoes collected in the respective control hut was 30.44% (− 1.142 -62.02) and 27.8% (14.86–40.75) for HM_open_ and MM_open_, respectively. These efficacy data suggest that 3D-WDST built with HM or MM cones had similar efficacies. Using baffles in the huts had no significant effect on the weekly mean female anopheline mosquito counts in the 3D-WDST of the HM_open_ vs. HM_baffles_ or the MM_open_ vs. MM_baffles_ (Table 6). HM and MM type cones also had no significant effect on the weekly mean female culicine mosquito count in the 3D-WDST of the HM_open_ vs. the MM_open_ condition, 4.83 (− 1.85 to 11.52) and 3.16 (− 1.50 to 7.83), respectively. The capturing efficacy of 3D-WDST in different hut conditions is summarized in Table 7.

**Table 7:** Capturing efficacy of 3D-WDST relative to the treatment hut and the control hut

	Capturing efficacy relative to the treatment hut			
	HM_open_ % (95% CI)	MM_open_ % (95% CI)	HM_baffles_ % (95% CI)	MM_baffles_ % (95% CI)
Total mosquitoes	25.07 (12–38.15)	29.34 (10–48.7)	22.11 (− 2.36–46.58)	49.58 (23.39–75.78)
Female	26.51 (9.65–43.37)	31.95 (8.96–54.94)	23.01 (− 3.35–49.36)	50.72 (19.79–81.65)
Anophelines				
Total	21.63 (9.79–33.46)	27.67 (10.23–45.1)	19.99 (− 4.71–44.68)	48.42 (21.33–75.51)
Female	24.44 (8.06–40.82)	30.55 (9.39–51.71)	21.06 (− 6.39–48.52)	50.19 (18.97–81.4)
Culicine				
Total	55.42 (12.16–98.68)	41.19 (− 4.89–87.26)	41.67 (− 9.92–93.26)	55.56 (2.88–10.2)
Female	27.43 (− 17.17–72.03)	47.22 (− 7.44–101.9)	50 (− 7.48–107.5)	38.89 (− 12.6–90.38)

	Capturing efficacy relative to control hut			
	HM_open_	MM_open_	HM_baffles_	MM_baffles_
Total mosquitoes	21.92 (4.83*–*39)	21.58 (9.48*–*33.66)	59.19 (− 79.32*–*197.7)	80.33 (− 26.3*–*187)
Female	27.17 (1.46*–*52.88)	25.08 (12.98*–*37.18)	69.56 (− 90.09*–*229.2)	95.36 (− 36.52*–*227.2)
Anophelines				
Total	21.13 (2.80*–*39.45)	22.82 (10.72*–*34.91)	58.31 (− 83.1*–*199.7)	84.13 (− 34.51*–*202.8)
Female	30.44 (− 1.14*–*62.02)	27.8 (14.86*–*40.75)	63.68 (− 88.71*–*216.1)	92.08 (− 32.41*–*216.6)
Culicine				
Total	53.53 (− 37.21*–*144.3)	16.49 (0.34*–*32.64)	66.67 (− 60.43*–*193.8)	66.67 (12.47*–*120.9)
Female	33.27 (− 45.32*–*111.9)	10.35 (− 2.36*–*23.06)	16.67 (− 26.18*–*59.51)	25 (− 18.9*–*68.9)

Meteorological conditions influence the relative abundance of various mosquito species by affecting the suitability and availability of breeding habitats. Trials I and II were conducted during the long rainy season in May–June of 2016 and 2017, respectively. This period was known for the prevalence of the malaria vector *An. gambiae* s.l. in the study area. In contrast, Trial III took place during the short rainy season in November–December 2017 when *An. funestus* s.l. was known to be more abundant than other malaria vector species.

Data from Trials I and II revealed that both *An. gambiae* s.l. and *An. funestus* s.l. were captured in the 3D-WDST in approximately equal numbers in both trials (Table 8). Moreover, an analysis of the total number of mosquitoes collected within each trial also indicated similar counts for both *An. gambiae* s.l. and *An. funestus* s.l. (Table 9).

**Table 8:** Total mosquitoes captured in the 3D-WDST across hut conditions in Trials I, II, and III

Trial	Row Labels	*Anopheles funestus* s.l.			*Anopheles gambiae* s.l.			Culicines			Grand Total
		Female	Male	Total	Female	Male	Total	Female	Male	Total	
Trial I	DC	8	2	10	5	8	13	23	5	28	51
	DO	28	10	38	48	8	56	29	5	34	128
	SC	0	0	0	1	3	4	1	0	1	5
	SO	2	7	9	6	1	7	2	0	2	18
	Grand Total	38	19	57	60	20	80	55	10	65	202
Trial II	T_open_	12	14	26	49	2	51	52	6	58	135
	T_baffles_	31	19	50	37	2	39	109	6	115	204
	Grand Total	43	33	76	86	4	90	161	12	173	339
Trial III	HM_open_	58	16	74	2	0	2	18	11	29	105
	HM_baffles_	26	4	30	0	0	0	3	2	5	35
	MM_open_	100	19	119	1	1	2	14	5	19	140
	MM_baffles_	59	16	75	0	0	0	3	2	5	80
	Grand Total	243	55	298	3	1	4	38	20	58	360

**Table 9:** Total mosquitoes collected across hut conditions in Trials I, II, and III

	*Anopheles funestus s.l*			*Anopheles gambiae s.l*			Culicines			Grand total
	Female	Male	Total	Female	Male	Total	Female	Male	Total	
Trial I	238	81	319	303	130	433	208	36	244	996
Trial II	242	302	544	365	37	402	723	60	783	1729
Trial III	1334	571	1905	22	4	26	109	47	156	2087

In contrast, data from Trial III demonstrated that during the November–December 2017 period, *An. funestus* s.l. emerged as the predominant malaria vector, with 1905 *An. funestus* s.l. specimens collected within the trial, compared to 26 *An. gambiae* s.l., as indicated in Table 9.

Notably, despite variations in species prevalence, the 3D-WDST's capturing efficiencies were not significantly different across the trials. This suggests that both mosquito species exhibited similar behaviors that made them equally susceptible to capture using the 3D-WDST.

## Discussion

The increasing prevalence of insecticide resistance in mosquito vectors has renewed global interest in the development of sustainable and non-insecticidal mosquito control alternatives. This study successfully assessed the efficacy of an insecticide-free, novel 3D-Screens in capturing wild mosquito vectors under semi-field conditions. Over the course of three experimental hut evaluations, we tested the 3D-Screens in two different 3D-WDST setups, one setup with a 3D-Screen and a traditional screen and the second with two 3D-Screens, both forming the window double-screen trap setup. Experimental hut conditions with open and closed eaves were also assessed. Moreover, we tested two different types of the cones used to create the 3D-Screens, cones that were made of screen mesh (traditional screen) and industrially manufactured plastic cones. The results of the three semi-field trials that took place in 2 successive years and covered long and short rainy seasons showed that the 3D-WDST setup made of two 3D-Screens was effective in capturing up to 51% (24–51% depending on the year and the rainy season) of the mosquitoes entering and escaping the experimental huts through the windows.

While window screening to control malaria is not new [27–33], the 3D-WDST concept stands out among other mosquito control methods because of its dual functionality as a house-proofing intervention as wells as a mosquito capturing tool. Additionally, the 3D-WDST setup with 3D-Screens on both sides facilitates mosquito capturing not only from outside (entry point) but also from inside (exit point) the house, adding a greater value to the intervention because of its ability to also capture mosquitoes exiting houses, through the windows, which could have been infected with the malaria parasite.

The trial results demonstrate a reasonable agreement with the laboratory study data, where the cone-based 3D-Screen prototype effectively captured a substantial percentage (92%) of the mosquitoes introduced into the wind tunnel when utilized in a double-screen configuration [14]. However, under semi-field conditions, the efficacy of mosquito capture was comparatively lower, reaching up to 51%. This decrease in efficacy can be attributed to the disparity in environmental conditions between a wind tunnel and a semi-field condition. In a wind tunnel, the mosquitoes are confined in proximity to the intervention and the luring agent, whereas in a semi-field condition, they roam freely in an open space.

The installation of the 3D-WDST in huts with open eaves led to a higher number of trapped mosquitoes compared to huts with closed eaves. This could suggest that some mosquitoes entered the huts through the eaves at night, escaped through the 3D-WDST in the morning, and got trapped, while in huts with closed eaves mosquitoes that were captured in the 3D-WDST were those that entered the huts only through the 3D-WDST. This indicates that both eaves and windows were mosquito entry points and the 3D-WDST could be more effective in controlling mosquito population when entry points such as eaves are left open. Additionally, the 3D-WDST setup featuring two 3D-Screens was notably more effective than the 3D-WDST configuration with only one 3D-Screen. This improved performance can be credited to the design of the setup, which enables mosquitoe trapping from both the exterior and interior of the house. Following this finding, in Trial II we explored the efficacy of the 3D-WDST with two 3D-Screens, creating a scenario where mosquitoes could freely enter the huts through open eaves but could not escape through them, allowing only window openings as exit point. This was achieved by introducing baffles (*T*_baffles_ treatment condition) in the eaves, which act as a funneling system for mosquito entry while blocking their escape. Although the capturing efficacy was higher for the *T*_baffles_ condition, the absolute count of the captured mosquitoes was similar in the 3D-WDST of the *T*_open_ and *T*_baffles_ conditions, suggesting that the enhancement of the calculated efficacy was due to fewer free mosquitoes collected in the hut spaces of the *T*_baffles_ condition possibly because of limited entry of mosquitoes through the baffles. This could indicate that blocking eaves as exit point had limited effect on the number of mosquitoes trapped in the 3D-WDST. On the other hand, the number of culicines was marginally higher, although statistically insignificant (*P* = 0.06), in the 3D-WDST of huts with baffles compared to those with open eaves. This could suggest that culicines tended to exit houses through eaves, and when eaves were blocked they tried to find other exit points such as window openings. It could also suggest that more culicines entered the huts through the eaves because of either their overall abundance or their persistence to enter the huts through the baffles, although the latter has to be proven in other studies. Based on these results, we decided to test an industrial design of 3D-Screens in the third trial and compared it with the design used in the first two trials. While the two versions of screens differed only in the material used, they performed equally in terms of their efficacy. The findings from the third trial allowed us to evaluate the design of the 3D-Screens for future large-scale implementation, which will require a cost-effective production process. The experimental hut studies did not assess the impact of environmental factors such as temperature, humidity, and rainfall on 3D-WDST. Additionally, the long-term integrity and durability of the 3D-WDST could not be assessed because of the limited duration of the studies. However, the 3D-Screens with industrially manufactured cones would likely be able to endure various field conditions because of the durable plastic materials used in the production of the cones.

In our study, we also observed a clear influence of meteorological conditions on the relative abundance of various mosquito species due to their impact on breeding habitat suitability and availability. Trials I and II, conducted during the long rainy seasons in May–June in 2016 and 2017, respectively, were characterized by the presence of both *An. gambiae* s.l. and *An. funestus* s.l. In contrast, Trial III took place during the short rainy season in November–December 2017, during which *An. funestus* s.l. was notably more abundant than other malaria vector species. Our findings from Trials I and II revealed a consistent pattern: both *An. gambiae* s.l. and *An. funestus* s.l. were captured in the 3D-WDST in nearly equal numbers during both trials. This pattern was further corroborated when analyzing the total mosquito counts within each trial, demonstrating that *An. gambiae* s.l. and *An. funestus* s.l. exhibited comparable numbers. However, the dynamics shifted in Trial III, conducted during the November–December 2017 period. Here, *An. funestus* s.l. emerged as the predominant malaria vector. Notably, what is of significance is that despite these variations in species prevalence, our results indicate that the capturing efficiencies of the 3D-WDST remained consistently robust across all trials. This suggests that both *An. gambiae* s.l. and *An. funestus* s.l. exhibited similar behaviors that rendered them equally susceptible to capture using the 3D-WDST. These insights underscore the versatility of the 3D-WDST as a valuable tool for potential effective malaria mosquito control irrespective of variations in species prevalence.

House screenings, which are common in developing countries, mostly to keep nuisance insects away, were associated with protection against malaria when implemented in mosquito control studies in endemic areas [34, 35]. A study in Magoda, Tanzania [36], examined the entomological impact of several modified housing designs and found a substantial reduction in indoor mosquitoes. A randomized controlled trial in Africa measured the clinical outcomes of house screening in an African setting and found that window and door screens and closed eaves halved the prevalence of anemia in children [33]. The benefit of making small changes to existing housing conditions such covering eaves with plywood and addition of netting and plastic screens has been demonstrated earlier in an experimental hut trial [31] in The Gambia where the house entry of principal malaria vector decreased substantially. While these approaches are effective in reducing house entry of disease vectors, widespread implementation of modifying housing in a resource-low setting might not be a practical option in terms of associated costs. An average cost of house screening per person in the study from The Gambia was $10 if additional netting material was made free, which is still much more expensive than the average $2 for LLINs [33, 35]. Similarly, the study in Tanzania with modified housing design presents several challenges in implementation on a local level and its cost-effectiveness [36]. The raw materials used in designing the house, such as bamboos to keep the relative humidity and temperature of the house lower, might sound ideal and practical, but on the local level, acquiring the timbers and bamboos locally to implement such design changes on a community level might not be entirely possible in terms of logistics, availability of resources, and cost-effectiveness. As for the 3D-Screens, the materials costs and other associated costs to produce a single 3D-WDST would be approximately $2 and $3 for the HM and MM 3D-Screens, respectively, making it a cost-effective alternative to be added on existing window openings or even while constructing the house itself, and the raw materials used in producing the 3D-Screens such as polyester nets, nails, and timbers can easily be sourced locally.

Considering the 3D-WDST setup is a more permanent option for mosquito control where the screens are placed on the window openings (by replacing the existing window setup), the intervention will likely receive a greater community acceptance because it provides a permanent solution to stop mosquito entry through windows and reduces the mosquito population in the surroundings. A similar entry trap, Lehmann’s funnel trap, has been tested in the field condition and has demonstrated significant efficacy in capturing mosquitoes as well [37, 38]. While the design concept and results from the field study suggest high efficacy of this trap system in capturing mosquitoes, the implementation of this kind of trap at the household level would be a less likely option given its bulky design. The appealing design of the 3D-WDST and its customizable installation on the other hand make it a visually appealing mosquito control tool and an attractive addition to community-based interventions, thereby enhancing its acceptance among the locals.

Current practice for indoor mosquito management in Africa mostly relies on usage of LLINs and IRS. Most houses in the countryside are locally made using mud bricks and timbers available within the surroundings. House-proofing against mosquitoes by closing eaves and other ports of mosquito entry means compromising the ventilation, and the practice is less favored. Qualitative studies conducted earlier have also shown that different aspects of personal comfort play important roles in the decision to use nets regularly. Barriers to comfort such as feeling uncomfortable, suffocation, feeling captured inside, perceived difficulty in breathing, or feeling itchy also influence the daily net usage [39–42]. The 3D-Screens therefore can serve as a good supplement to LLINs in controlling malaria transmission because the 3D-WSDT does not compromise ventilation, can be produced locally, and is also cost-effective. The next step of evaluation would be phase III community studies where the epidemiological and entomological impact of 3D-Screens at community levels will be evaluated. The community acceptance of this novel mosquito control method, community engagement, and its cost-effectiveness in terms of production and management as well as screen integrity and durability will also be evaluated during the prospective phase III studies.

## Data Availability

All datasets supporting the conclusion of this study is included within the paper.
